# Causal effects of lipid-lowering drugs on skin diseases: a two-sample Mendelian randomization study

**DOI:** 10.3389/fmed.2024.1396036

**Published:** 2024-09-25

**Authors:** Yong Liu, Hui Liu, Queqiao Bian

**Affiliations:** ^1^Tianjin Key Laboratory of Extracorporeal Life Support for Critical Diseases, Department of Dermatology and STD, The Third Central Hospital of Tianjin, Artificial Cell Engineering Technology Research Center, Tianjin Institute of Hepatobiliary Disease, Tianjin, China; ^2^Tianjin Institute of Hepatobiliary Disease, The Third Central Hospital of Tianjin, Tianjin, China

**Keywords:** Mendelian randomization study, single-nucleotide polymorphisms, genome-wide association studies, lipid-lowering drugs, psoriasis, melanoma skin cancer, nonmelanoma skin cancer

## Abstract

**Background:**

Although previous studies have indicated an association between low-density lipoprotein (LDL) and skin diseases, their causal effects remain inconclusive. This study aimed to assess the causal relationship between genetically proxied lipid-lowering drugs and skin cancers and psoriasis.

**Methods:**

Two-sample Mendelian randomization (MR) analysis was performed using single-nucleotide polymorphisms (SNPs) from genome-wide association studies (GWAS). The inverse-variance weighted (IVW) method was used to determine causal relationships. The “leave-one-out” sensitivity test, Cochran’s Q-statistic and MR-Egger intercept were used to assess heterogeneity and horizontal pleiotropy.

**Results:**

We identified 3-hydroxy-3-methylglutaryl-coenzyme A reductase (HMGCR) and proprotein convertase subtilisin-kexin type 9 (PCSK9) as genetically proxied lipid-lowering drugs. Genetically proxied inhibition of HMGCR (stains) was causally associated with reduced risk of nonmelanoma skin cancer (OR 0.982, 95% CI 0.967–0.997, *p* = 0.016 by weighted median; OR 0.977, 95% CI 0.966–0.989, *p* < 0.001 by IVW) and psoriasis (OR 0.585, 95% CI 0.378–0.905, *p* = 0.016 by IVW), while PCSK9 inhibition (alirocumab) was causally associated with reduced risk of psoriasis (OR 0.560, 95% CI 0.413–0.761 by weighted median; OR 0.564, 95% CI 0.447–0.712 by IVW; *p* < 0.001) in the ieu-b-5089 dataset. Similar results were observed in the ieu-b-110 dataset for HMGCR and PCSK9. Sensitivity analysis revealed no evidence of heterogeneity or horizontal pleiotropy.

**Conclusion:**

This study revealed the existing HMGCR inhibitors (stains) might be protective for reducing nonmelanoma skin cancer risk, and HMGCR inhibitors (stains) and PCSK9 inhibitor (alirocumab) might be promising for reducing psoriasis risk in the European population.

## Introduction

The skin is the largest organ of the body and serves as a barrier to protect from external harm. Skin diseases are a major public health issue, accounting for 1.79% of the global disease burden, and are expressed as disability-adjusted life years (DALYs) (1). Melanoma and nonmelanoma skin cancers, such as basal cell carcinoma (BCC) and squamous cell carcinoma (SCC), are the most frequently diagnosed malignant skin diseases and are associated with high medical costs and high mortality rates (2). Patients with melanoma and nonmelanoma skin cancer may benefit from immunotherapy and targeted therapy (3), however, the 5-year survival of melanoma patients remains unsatisfactory (4–6). Psoriasis is a complex, chronic, systemic, immune-mediated disease with a prevalence of 0.51–11.43% in adults and 1.37% in children (7). It is thought to be as an immune-mediated genetic disorder (8), and it may predispose individuals to psoriatic arthritis and is associated with an increased risk of skin cancer (9). Although systemic therapy for psoriasis has improved dramatically, there is still a need to find a cure for complex psoriasis (10). Notably, the use of biologics in patients with psoriasis increases the risk of melanoma and nonmelanoma skin cancers (11). Therefore, it is necessary to discover promising targets for the treatment of melanoma skin cancer, nonmelanoma skin disease and psoriasis.

Low-density lipoprotein (LDL) is an important extracellular lipoprotein that carries cholesterol. LDL cholesterol (LDL-C) represents the total cholesterol content of LDL particles, and an elevated level of LDL-C is the key factor associated with an increased risk of atherosclerotic cardiovascular disease (12). A previous study revealed positive associations of intermediate-density lipoprotein, very low LDL and low LDL lipids with the risk of distal colon cancer (13). In addition, there is a causal relationship between LDL-C levels and the risk of hepatocellular carcinoma mediated by coronary artery disease (14). Moreover, the accumulation of oxidized LDL has been observed in psoriatic skin lesions (15). The LDL score and LDL particle size in psoriatic arthritis patients are associated with carotid intima-media thickness (16). Elevated level of LDL-C may be a risk factor for atopic dermatitis in Korean adolescents (17). These findings may indicate that changes in LDL-C may be implicated in the development of skin diseases. However, the effects of LDL-C on melanoma skin cancer, nonmelanoma skin disease and psoriasis remain poorly understood.

Two-sample Mendelian randomization (MR) analysis is a method for estimating the causal effect of an exposure on an outcome using generalized genome-wide association studies (GWAS) data (18). Two-sample MR analyses have been widely applied to estimate the causal effects of lipid-lowering drugs on different fields of public issues, such as systemic lupus erythematosus (19), psoriasis (20), gout (21), osteoarthritis (22), and inflammatory bowel disease (23). These results may facilitate lipid-lowering drug repurposing and provide promising targets for disease management. Currently, evidence of the potential causal effects of lipid-lowering drugs on melanoma skin cancer, nonmelanoma skin disease and psoriasis using two-sample MR analysis is still lacking. In the present study, we therefore performed a two-sample MR analysis to reveal the potential causal relationship between genetically proxied lipid-lowering drugs and three skin diseases, including melanoma skin cancer, nonmelanoma skin cancer and psoriasis.

## Materials and methods

### Data sources

Given that lipid-lowering drugs typically lower cholesterol and triglyceride levels by affecting low-density lipoprotein-cholesterol (LDL-C), LDL-C was selected as the exposure data for this study. We downloaded the LDL-C genetic dataset from the MRC Integrative Epidemiology Unit (IEU) GWAS^1^ (24), a manually curated collection of complete GWAS summary datasets. Moreover, GWAS summary statistics for skin-related diseases such as melanoma skin cancer, nonmelanoma skin cancer and psoriasis were also obtained from IEU GWAS. Table 1 lists the GWAS data information for the exposure data and outcome data. In this study, the ieu-b-5089 dataset was used as a discovery dataset, and the ieu-b-110 dataset was used as a validation dataset.

**Table 1:** GWAS data of exposure data and outcome data.

Characteristics	Data number	Types	Data sources	Sample size	Ancestry	Number of SNPs	Author	Year
LDL-C	ieu-b-5089	exposure data	UK Biobank	201,678	European	12,321,875	Si Fang	2022
LDL-C	ieu-b-110	exposure data	UK Biobank	440,546	European	12,321,875	Richardson, Tom	2020
Melanoma skin cancer	ieu-b-4969	outcome data	UK Biobank	375,767	European	11,396,019	Burrows	2021
Non-melanoma skin cancer	ieu-b-4959	outcome data	UK Biobank	395,710	European	12,321,875	Burrows	2021
Psoriasis	ebi-a-GCST90018907	outcome data	NA	483,174	European	24,191,364	Sakaue S	2021

### Determination of genetic instrumental variables

We predicted the target genes for various lipid-lowering drugs through the DrugBank database^2^ (25), a comprehensive, free-to-access, online database containing information on drugs and drug targets. The genetic IVs for MR analysis was constrained by three assumptions. First, the single nucleotide polymorphisms (SNPs) selected in this study had to be strongly associated with exposure. SNPs associated with LDL-C at the genome-wide significance threshold (*p* < 5 × 10^−8^) were selected for analysis. Second, linkage disequilibrium (LD) was performed, and SNPs with LD cutoff value of *r*^2^ < 0.3, allele frequency > 0.01, and located within 100 kb upstream or downstream of target genes were selected (26, 27). In addition, to ensure that our results were unlikely to be biased by weak instruments, IVs with F-statistics <10 were eliminated from the analysis (28). Meanwhile, MR-PRESSO was used to assess the bias of each SNP, in order to eliminate the SNPs with bias. After screening, the SNPs of HMGCR and PCSK9 were ultimately retained.

### Two-sample MR analysis

MR-Egger, inverse-variance weighting (IVW), the weighted median, and the weighted mode are commonly used methods for two-sample MR analysis (29–31). IVW is a fundamental and commonly used method that can obtain unbiased estimates of the status without horizontal pleiotropy. Therefore, in this study, the IVW method was used to determine the causal relationship between genetically proxied lipid-lowering drugs and skin diseases using the TwoSampleMR R package. Additionally, the MR-Egger method was used in the present study to improve the reliability of the findings (32). The two-sample MR analysis was performed based on LDL-C data as the exposure, disease as the outcome, and selected SNPs of target genes.

### Sensitivity analysis

To evaluate the robustness of the MR analysis, a sensitivity test was conducted using the “leave-one-out” method, in which one SNP was removed at a time and IVW was conducted based on the remaining SNPs. Cochran’s Q-statistic was used to evaluate heterogeneity using the MR-Egger and IVW methods, and a *p* value <0.05 was regarded as indicating significant heterogeneity. Funnel plots were generated to assess publication bias and directional horizontal pleiotropy. The MR-Egger intercept was calculated to test for the presence of horizontal pleiotropy (judged at *p* < 0.05) (33).

### Statistics

All analyses were conducted using the “TwoSampleMR” package in R (version 4.1.2, https://www.rproject.org/). The results are presented as *β* values and 95% confidence intervals (CIs). A *p* value <0.05 was regarded as statistically significant.

## Results

### Selection of IVs for MR analysis

By searching the target genes of lipid-lowering drugs in the DrugBank database, we found that 3-hydroxy-3-methylglutaryl-coenzyme A reductase (HMGCR), dipeptidyl peptidase 4 (DPP4), aryl hydrocarbon receptor (AHR), histone deacetylase 2 (HDAC2), and nuclear receptor subfamily 1, Group I, member 3 (NR1I3) were target genes for atorvastatin; proprotein convertase subtilisin-kexin type 9 (PCSK9) was found to be a target gene for alirocumab; HMGCR and HDAC2 were identified as target genes for fluvastatin and pravastatin sodium; HMGCR, integrin subunit alpha L (ITGAL), and HDAC2 were identified as target genes for lovastatin and simvastatin; and HMGCR and ITGAL were found to be target genes for rosuvastatin (Supplementary Table 1). Next, we screened the SNPs of all the predicted target genes and retained only those of HMGCR and PCSK9 for further analysis. In the ieu-b-5089 dataset, a total of seven SNPs, including rs10079346, rs17648121, rs2006760, rs2303152, rs4704213, rs55727654, and rs6453131 were identified in HMGCR, and 16 SNPs (rs10493176, rs11587071, rs11591147, rs12732125, rs17111503, rs2483205, rs2495500, rs3976734, rs472495, rs4927191, rs505151, rs530804537, rs6691964, rs72660539, rs7543163, and rs77875082) were identified in PCSK9 for melanoma skin cancer (Supplementary Tables 2, 3), nonmelanoma skin cancer (Supplementary Tables 4, 5), and psoriasis (Supplementary Tables 6, 7). In the ieu-b-110 dataset, we identified 19 SNPs in HMGCR and 33 SNPs in PCSK9 for the three skin diseases (Supplementary Tables 8–13). The F-statistic of all IVs was greater than 10, which indicated that our results were unlikely to be biased by weak IVs. In addition, MR-PRESSO was used to assess the bias of SNPs, and no SNPs showing significant bias were detected in this study.

### Two-sample MR analysis

**Figure 1:**
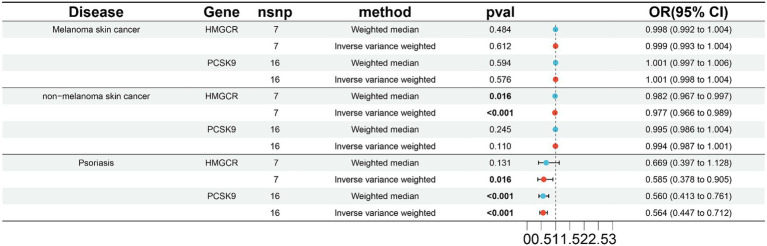
Results of two-sample MR analysis between lipid-lowering drugs and skin diseases in the ieu-b-5089 dataset. The weighted median and inverse-variance weighted (IVW) methods are used for two-sample MR analysis. HMGCR, 3-hydroxy-3-methylglutaryl-coenzyme A reductase; PCSK9, Proprotein convertase subtilisin-kexin type 9; OR, Odds ratio; CI, Confidence interval. The *p* value <0.05 is considered statistically significant.

According to the results of two-sample MR analysis of the ieu-b-5089 dataset (Figure 1; Supplementary Figure 1), there was no significant association between HMGCR or PCSK9 and melanoma skin cancer. However, genetically proxied inhibition of HMGCR had a significant association with reduced risk of nonmelanoma skin cancer (OR 0.982, 95% CI 0.967–0.997, *p* = 0.016 by weighted median; OR 0.977, 95% CI 0.966–0.989, *p* < 0.001 by IVW), but there was no significant causal association between PCSK9 and nonmelanoma skin cancer. Based on the IVW method, genetically proxied inhibition of HMGCR was notably causally associated with reduced risk of psoriasis (OR 0.585, 95% CI 0.378–0.905, *p* = 0.016), and genetically proxied inhibition of PCSK9 was significantly associated with reduced risk of psoriasis based on the weighted median and IVW methods (OR 0.560, 95% CI 0.413–0.761; OR 0.564, 95% CI 0.447–0.712; *p* < 0.001).

### Independent data validation

**Figure 2:**
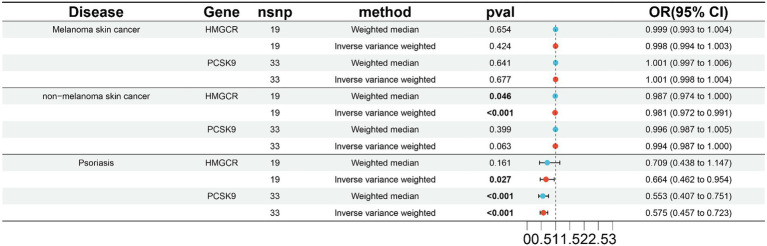
Validation of two-sample MR analysis in the ieu-b-110 dataset. The weighted median and inverse-variance weighted (IVW) methods are used for two-sample MR analysis. HMGCR, 3-hydroxy-3-methylglutaryl-coenzyme A reductase; PCSK9, Proprotein convertase subtilisin-kexin type 9; OR, Odds ratio; CI, Confidence interval. The *p* value <0.05 is considered statistically significant.

To validate the results in the ieu-b-5089 dataset, the independent exposure dataset ieu-b-110 with the above three skin diseases was subjected to MR analysis, with 19 SNPs in HMGCR and 33 SNPs in PCSK9 as IVs. As displayed in Figure 2 and Supplementary Figure 2, HMGCR and PCSK9 had no significant causal effects on melanoma skin cancer based on the weighted median and IVW methods. Genetically proxied reduction in HMGCR had a significant causal effect on the reduced risk of nonmelanoma skin cancer according to the weighted median (OR 0.987, 95% CI 0.974–1.000, *p* = 0.046) and IVW (OR 0.981, 95% CI 0.972–0.991, *p* < 0.001) methods. In addition, both inhibition of HMGCR (OR 0.664, 95% CI 0.462–0.954, *p* = 0.046 by IVW) and PCSK9 (OR 0.553, 95% CI 0.407–0.751 by weighted median; OR 0.575, 95% CI 0.457–0.723 by IVW; *p* < 0.001) was significantly associated with reduced risk of psoriasis.

### Sensitivity analysis

**Figure 3:**
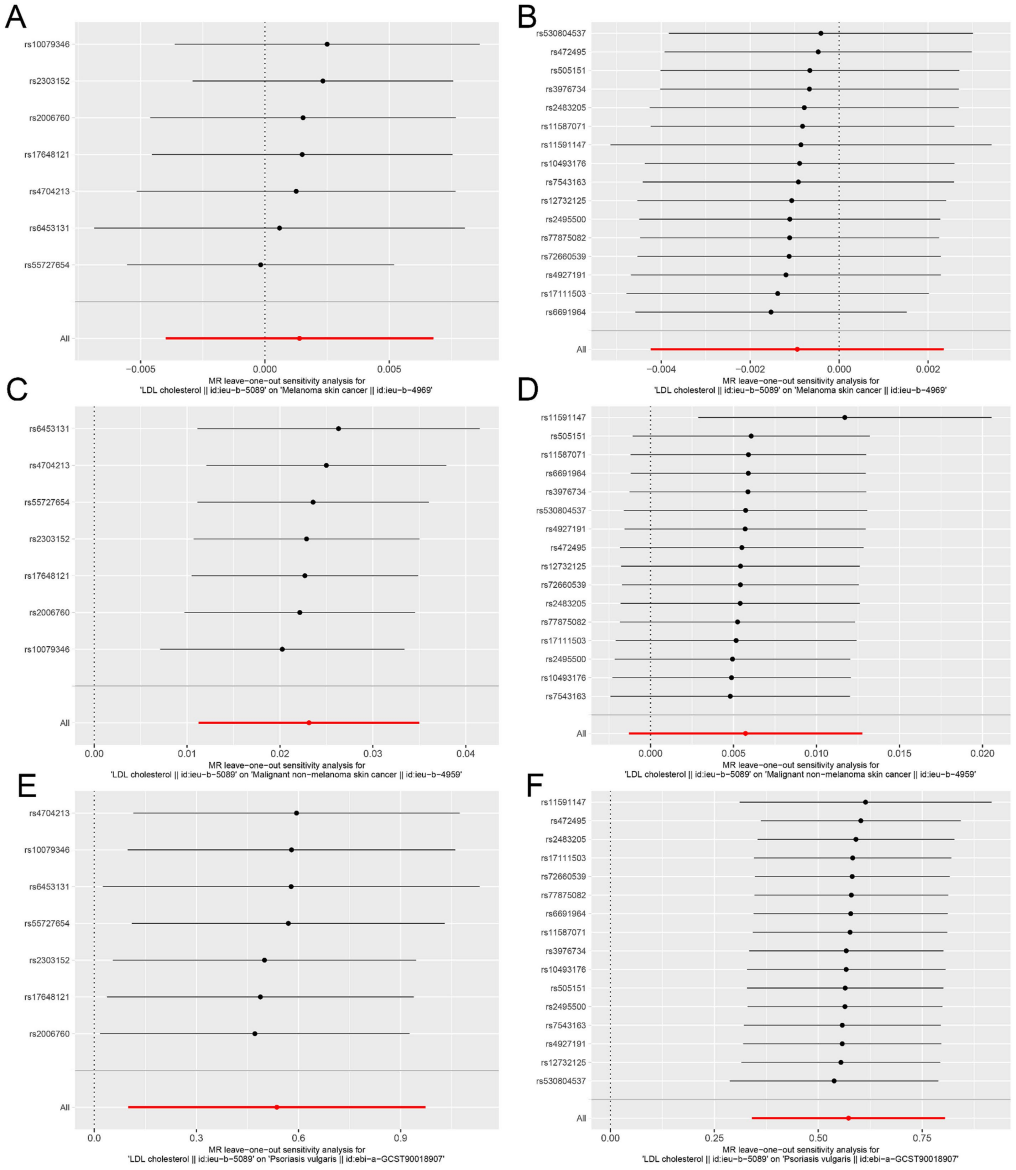
Sensitivity analysis assesses the influence of SNPs on the results of MR analysis using leave-one-out analysis in the ieu-b-5089 dataset. **(A)** Leave-one-out analysis for HMGCR on melanoma skin cancer (ieu-b-4969). **(B)** Leave-one-out analysis for PCSK9 on melanoma skin cancer (ieu-b-4969). **(C)** Leave-one-out analysis for HMGCR on nonmelanoma skin cancer (ieu-b-4959). **(D)** Leave-one-out analysis for PCSK9 on nonmelanoma skin cancer (ieu-b-4959). **(E)** Leave-one-out analysis for HMGCR on psoriasis (ebi-a-GCST90018907). **(F)** Leave-one-out analysis for PCSK9 on psoriasis (ebi-a-GCST90018907). The significance of red line is MR result.

The “leave-one-out analysis” was performed for each outcome to assess the stability of the results in the ieu-b-5089 dataset after removing a particular IV. As shown in Figures 3A,B, the overall error line did not change significantly, and all the error lines were located to the right of zero or to the left of zero. The results revealed that the difference between the MR results estimated by the other IVs and the total results was not significant after removing each SNP, which indicated that the MR results for HMGCR and PCSK9 inhibition in melanoma skin cancer were robust. After removing each SNP, the overall error line for nonmelanoma skin cancer did not change significantly (Figures 3C,D). Robust MR results were found for HMGCR and PCSK9 inhibition in psoriasis (Figures 3E,F).

**Figure 4:**
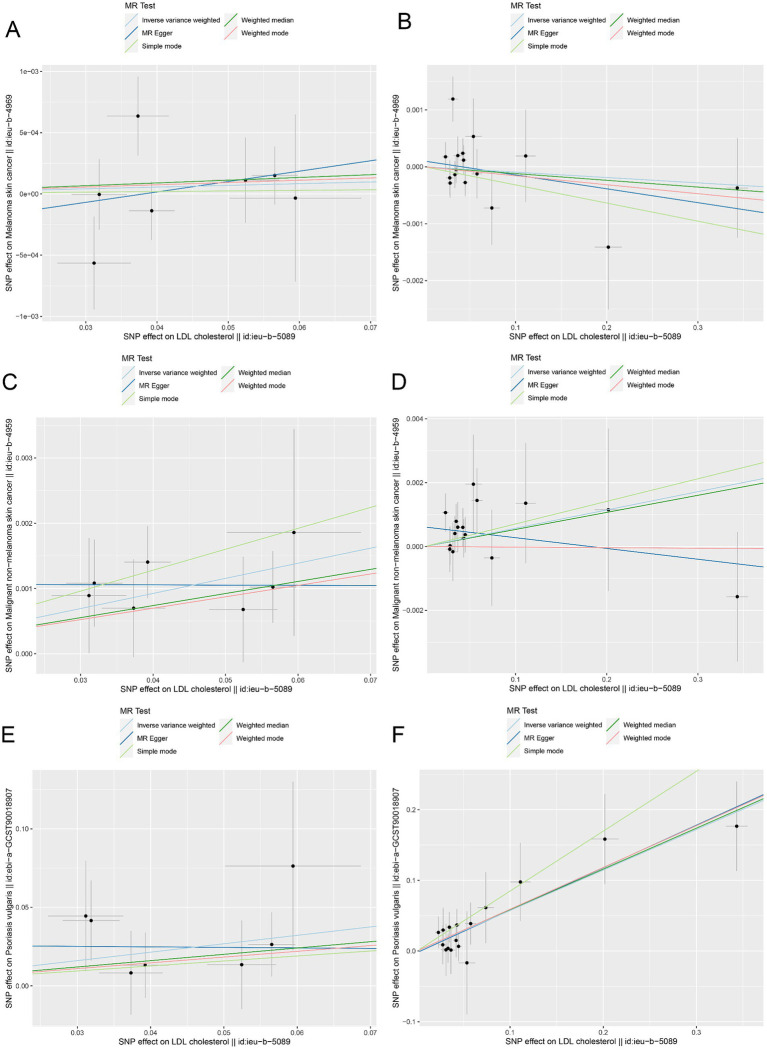
The MR effect scatter plots of genetic associations between LDL-C and outcomes in the ieu-b-5089 dataset. **(A)** The MR effect scatter plots of genetic associations between HMGCR and melanoma skin cancer (ieu-b-4969). **(B)** The MR effect scatter plots of genetic associations between PCSK9 and melanoma skin cancer (ieu-b-4969). **(C)** The MR effect scatter plots of genetic associations between HMGCR and nonmelanoma skin cancer (ieu-b-4959). **(D)** The MR effect scatter plots of genetic associations between PCSK9 and nonmelanoma skin cancer (ieu-b-4959). **(E)** The MR effect scatter plots of genetic associations between HMGCR and psoriasis (ebi-a-GCST90018907). **(F)** The MR effect scatter plots of genetic associations between PCSK9 and psoriasis (ebi-a-GCST90018907). The slopes of each line represent the causal relationship for each method. The light blue line represents the inverse-variance weighted (IVW) method, the dark blue line represents the MR-Egger method, the dark green line represents the weighted median estimate, the light green line represents the Simple mode method, and the red line represents the weighted mode method.

Moreover, the MR effect scatter plots were generated using several methods, including the IVM, MR-Egger, simple mode, weighted median, and weighted mode. The results indicated a consistent trend in the predicted regression lines of HMGCR and PCSK9 for melanoma skin cancer (Figures 4A,B), nonmelanoma skin cancer (Figures 4C,D), and psoriasis (Figures 4E,F). MR-Egger regression showed no evidence of the presence of horizontal pleiotropy (*p* > 0.05) (Table 2), which suggested that the results were robust and not influenced by potential confounding pathways.

**Table 2:** The results of horizontal pleiotropy in the ieu-b-5089 dataset.

Genes	Outcomes	Egger_intercept	Standard error	*p* value
HMGCR	Melanoma skin cancer	−0.000323413	0.00056894	0.594309434
	Nonmelanoma skin cancer	0.001069842	0.001182496	0.407086199
	Psoriasis	0.026161665	0.044381038	0.581167381
PCSK9	Melanoma skin cancer	0.000100741	0.000143291	0.493548318
	Nonmelanoma skin cancer	0.000613011	0.000300539	0.060716101
	Psoriasis	−0.002346531	0.010738367	0.830178514

**Figure 5:**
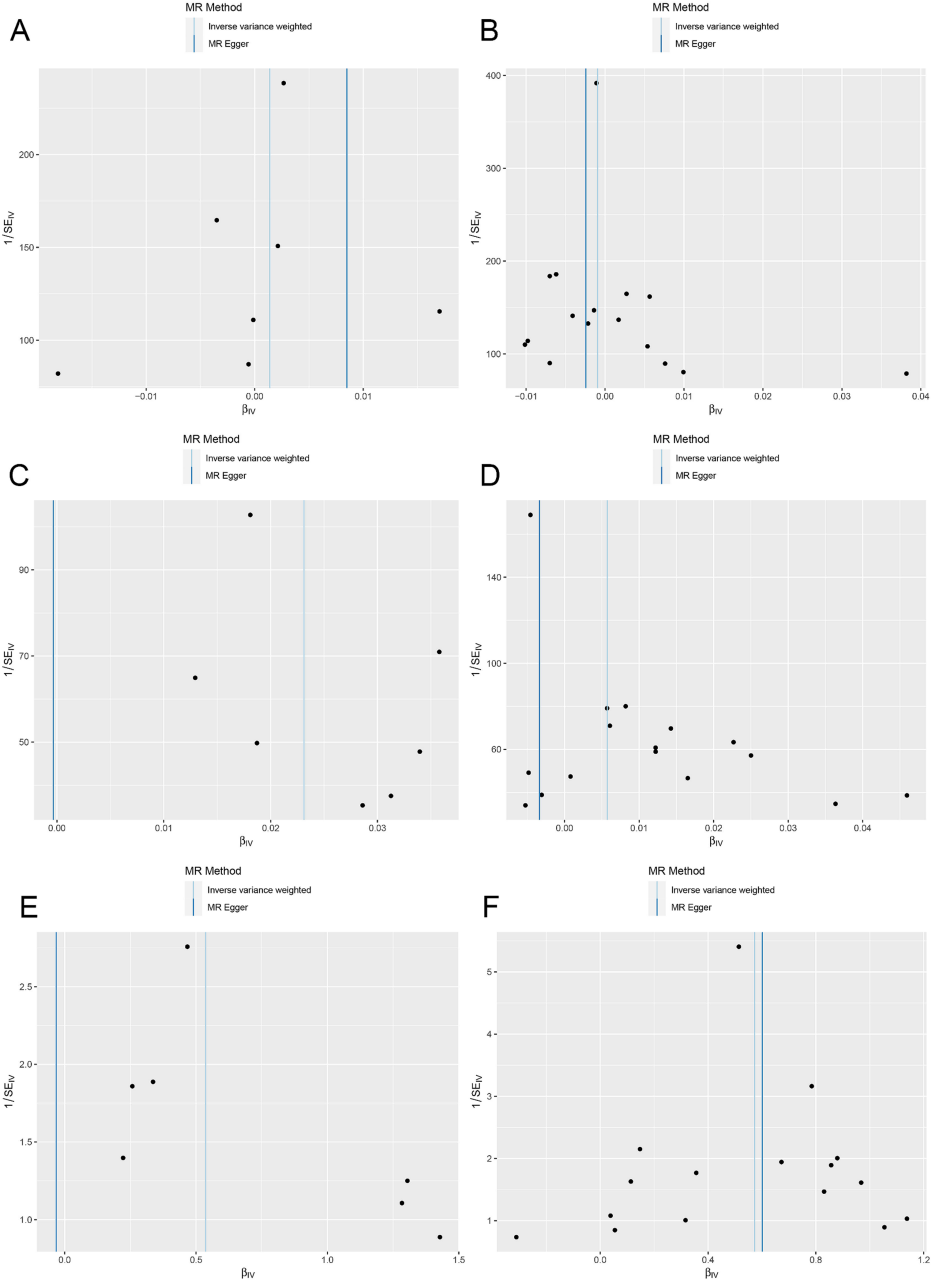
Funnel plots assess heterogeneity in the ieu-b-5089 dataset. **(A)** Funnel plots for heterogeneity among SNPs of HMGCR on melanoma skin cancer (ieu-b-4969). **(B)** Funnel plots for heterogeneity among SNPs of PCSK9 on melanoma skin cancer (ieu-b-4969). **(C)** Funnel plots for heterogeneity among SNPs of HMGCR on nonmelanoma skin cancer (ieu-b-4959). **(D)** Funnel plots for heterogeneity among SNPs of PCSK9 on nonmelanoma skin cancer (ieu-b-4959). **(E)** Funnel plots for heterogeneity among SNPs of HMGCR on psoriasis (ebi-a-GCST90018907). **(F)** Funnel plots for heterogeneity among SNPs of PCSK9 on psoriasis (ebi-a-GCST90018907). The light blue line represents the inverse-variance weighted (IVW) method and the dark blue line represents the MR-Egger method.

Additionally, heterogeneity was assessed using Cochran’s Q, which showed no evidence of significant heterogeneity (Table 3). Furthermore, funnel plots were generated to assess the heterogeneity of SNPs. The points on the left and right sides of the IVW line were symmetrically distributed, which indicated no significant evidence of heterogeneity for SNPs in HMGCR and PCSK9 for melanoma skin cancer (Figures 5A,B). Funnel plots of the SNPs in HMGCR and PCSK9 did not reveal significant asymmetry for SNPs for nonmelanoma skin cancer (Figures 5C,D) or psoriasis (Figures 5E,F) patients.

**Table 3:** The results of heterogeneity in the ieu-b-5089 dataset.

Genes	Outcomes	Methods	Q	Q_df	Q_p value
HMGCR	Melanoma skin cancer	MR Egger	6.23367166	5	0.28414113
		IVW	6.636533023	6	0.355771228
	Nonmelanoma skin cancer	MR Egger	1.139847393	5	0.950516366
		IVW	1.958388415	6	0.923485528
	Psoriasis	MR Egger	2.527235342	5	0.772387905
		IVW	2.87472018	6	0.824398583
PCSK9	Melanoma skin cancer	MR Egger	17.12949114	14	0.249342924
		IVW	17.7342664	15	0.276893578
	Nonmelanoma skin cancer	MR Egger	6.309210424	14	0.958085774
		IVW	10.46959963	15	0.789208115
	Psoriasis	MR Egger	4.837210697	14	0.987951651
		IVW	4.884961003	15	0.993056403

## Discussion

In the present study, a two-sample MR analysis was performed to reveal the potential causal effects of LDL-C on the risk of skin diseases including melanoma skin cancer, nonmelanoma skin cancer and psoriasis. HMGCR and PCSK9 were identified as potential target genes for lipid-lowering drugs, 7 SNPs were identified in the HMGCR gene, and 16 SNPs were identified in PCSK9 in the ieu-b-5089 dataset as IVs. MR analysis revealed that inhibition of HMGCR had a causal association with reduced risk of nonmelanoma skin cancer, and inhibition of HMGCR and PCSK9 was associated with reduced risk of psoriasis. To validate the results, 19 SNPs in HMGCR and 33 SNPs in PCSK9 screened in the ieu-b-110 dataset were used as IVs, and similar results were observed. Further sensitivity analysis revealed no evidence of the presence of horizontal pleiotropy or heterogeneity. The results provided more causal evidence for genetically proxied inhibition of HMGCR (stains) in reducing the risk of nonmelanoma skin cancer and psoriasis, and PCSK9 inhibition (alirocumab) in reducing the risk of psoriasis.

Proprotein convertase subtilisin-kexin type 9 is an important gene that encodes a protein belonging to the subtilisin-like proprotein convertase family. The encoded protein is expressed in hepatic, intestinal and renal tissues and escorts specific receptors for lysosomal degradation. The PCSK9-encoded protein is crucial for cholesterol and fatty acid metabolism. Previous observational studies have indicated a role for PCSK9 in skin diseases. For instance, the K14-Rac1V12−/+ psoriasis mice exhibit skin-specific PCSK9 expression, and the expression of PCSK9 correlates with impaired endothelial vascular health in psoriasis patients (34). Frątczak A has revealed that psoriasis patients with cyclosporine respond have decreased serum levels of NGAL while the serum level of PCSK9 is associated with total cholesterol and LDL at baseline and after 3 months of treatment, suggesting a role for PCSK9 in the treatment of dyslipidemia in psoriasis patients (35). Notably, recent studies have revealed the role of lipid-lowering drugs targeting PCSK9 in skin diseases. Wu demonstrated that the inhibition of PCSK9 (evolocumab) is causally associated with a reduced risk of systemic lupus erythematosus in a population of European ancestry (19). Zhao and colleagues have conducted a two-sample MR analysis using SNPs associated with LDL within ±100 kb of the HMGCR, NPC1L1 and PCSK9, and they have found that genetically proxied PCSK9 inhibition has a causal relationship with a lower risk of psoriasis, which is independent of circulating LDL levels; additionally, there is no causal relationship between HMGCR and psoriasis risk (20). The aforementioned MR analysis only demonstrated a causal effect of PCSK9 inhibition on psoriasis, but did not reveal a causal effect of HMGCR on skin diseases. In the present study, we not only indicated a causal effect of PCSK9 inhibition (alirocumab) on reduced risk of psoriasis, but also revealed a causal effect of HMGCR inhibition (stains) on both nonmelanoma skin cancer and psoriasis. The discrepancy in the causal effect of HMGCR on psoriasis should be investigated in the future.

The HMGCR is a key rate-limiting enzyme for cholesterol biosynthesis, and HMGCR is regulated by a negative feedback mechanism mediated by sterols and nonsterol metabolites derived from mevalonate, the product catalyzed by reductase. HMGCR has been used as a target for statins in the treatment of cardiovascular disease (36). A previous study has indicated that PPARδ and HMGCR are positively correlated with cholesterol content and fatty acid levels in the skin of obese individuals, and are associated with functional impairment of the skin barrier (37). Compelling evidence suggests the inhibitory effect of HMGCR as an anticancer target of physapubenolide on melanoma cells (38). Atorvastatin, an inhibitor of HMGCR, in combination with the electron transport chain complex I inhibitor IACS-010759, is a promising approach for inducing fatty acid metabolism-dependent regression of BRAF inhibitor-resistant melanomas (39). A recent study has suggested that statins, the HMGCR inhibitors, may be involved in the suppression of PGC1α expression in BRAF-inhibitor resistant melanomas (40). Nevertheless, evidence from randomized controlled trials supporting the benefit of lipid-lowering drugs targeting HMGCR in skin cancers is still lacking. In this study, two-sample MR analysis demonstrated for the first time a causal effect of HMGCR inhibition (stains) on reduced risk of nonmelanoma skin cancer and psoriasis, with no evidence of horizontal pleiotropy or heterogeneity. Our study might provide stronger evidence than traditional observational studies.

In the present study, we considered that lipid-lowering drugs decreased the levels of cholesterol and triglycerides by affecting LDL-C; therefore, we predicted target genes for lipid-lowering drugs in the DrugBank database and focused on all SNPs associated with LDL-C. In addition, SNPs associated with LDL-C were downloaded from the IEU GWAS, and the application of GWAS data allowed the identification of numerous strong associations for a wide range of phenotypes and diseases to infer potential causal links between exposure factors and outcomes (41). To validate the robustness of our results, validation was conducted in an independent dataset, and similar results were replicated. Hence, the aforementioned factors may contribute to the strength of the findings in this study.

In contrast to previous observational studies, we conducted drug-targeted MR analysis to evaluate the causal relationship between lipid-lowering drugs and three skin diseases. When the IV assumptions were met, MR was used to analyze the causal effect of exposures on outcomes with less bias from unmeasured confounders. However, some limitations should be noted in this study. First, the inclusion of skin cancer in this study is limited, as skin cancers include SCC, BCC and Kaposi’s sarcoma, as well as chronic psoriatic disease, including common psoriasis, psoriatic arthritis or other types. Thus, the causal association between HMGCR/PCSK9 and a specific type of disease needs further validation. Second, data obtained from the United Kingdom Biobank may lead to data overlap, which may have a certain impact on the analysis results. In addition, the data in this study were obtained from populations of European ancestry, and the application of lipid-lowering drugs inhibiting HMGCR and PCSK9 should be verified in other populations. We selected SNPs with LD cutoff value of *r*^2^ < 0.3, allele frequency > 0.01, and located within 100 kb upstream or downstream of target genes to generate IVs, while, the more relaxed clumping threshold might include more SNPs to ensure statistical power, which might bring some impacts on the results of the analysis. Moreover, further researches are necessary to elucidate the underlying mechanisms and potential therapeutic applications of HMGCR inhibitors (stains) in nonmelanoma skin cancer and psoriasis, and PCSK9 inhibitors (alirocumab) in psoriasis.

In conclusion, we demonstrated a causal effect of HMGCR inhibition on reduced risk of nonmelanoma skin cancer. HMGCR and PCSK9 inhibition was causally associated with reduced risk of psoriasis. This study provides novel directions for the management of nonmelanoma skin cancer and psoriasis.

## Data Availability

The datasets presented in this study can be found in online repositories. The names of the repository/repositories and accession number(s) can be found in the article/Supplementary material.

## Footnotes

^1^ https://gwas.mrcieu.ac.uk/

^2^ https://go.drugbank.com/

## Supplementary material

SUPPLEMENTARY FIGURE 1 Two-sample MR analysis using inverse-variance weighted (IVW) method in the ieu-b-5089 dataset. **(A,B)** MR effect size for lipid-lowering drug target genes HMGCR and PCSK9 on melanoma skin cancer (ieu-b-4969). **(C,D)** MR effect size for HMGCR and PCSK9 on nonmelanoma skin cancer (ieu-b-4959). **(E,F)** MR effect size for HMGCR and PCSK9 on psoriasis (ebi-a-GCST90018907). The black dots and lines represent the total estimate and 95% CI of MR analysis by MREgger regression and inverse-variance weighted (IVW) methods. The significance of red lines are MR results of MR-Egger and inverse-variance weighted (IVW) methods.

SUPPLEMENTARY FIGURE 2 Validation of the results from the two-sample MR analysis using inverse variance weighted (IVW) method in the ieu-b-110 dataset. **(A,B)** MR effect size for HMGCR and PCSK9 on melanoma skin cancer (ieu-b-4969). **(C,D)** MR effect size for HMGCR and PCSK9 on nonmelanoma skin cancer (ieu-b-4959). **(E,F)** MR effect size for HMGCR and PCSK9 on psoriasis (ebi-a-GCST90018907). The black dots and lines represent the total estimate and 95% CI of MR analysis by MR-Egger regression and IVW methods. The significance of red lines are MR results of MR-Egger and inverse-variance weighted (IVW) methods.
