# Supplementary figures and images for: Causal effects of lipid-lowering drugs on skin diseases: a two-sample Mendelian randomization study
Source: Front Med (Lausanne). 2024 Sep 25;11:1396036. doi: 10.3389/fmed.2024.1396036 (PMC11461303; doi:10.3389/fmed.2024.1396036)

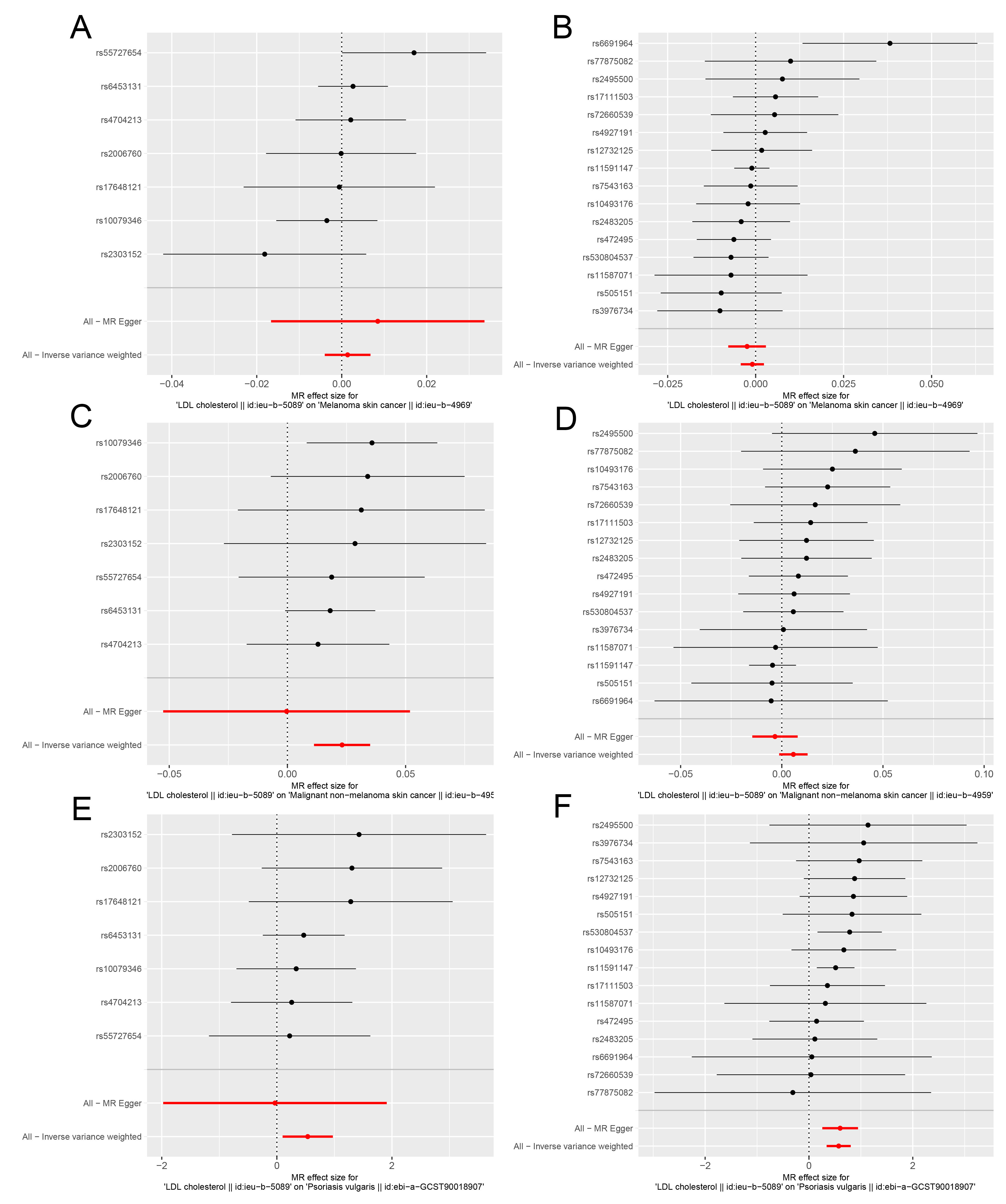

Supplement: SUPPLEMENTARY FIGURE 1 — Two-sample MR analysis using inverse-variance weighted (IVW) method in the ieu-b-5089 dataset. (A,B) MR effect size for lipid-lowering drug target genes HMGCR and PCSK9 on melanoma skin cancer (ieu-b-4969). (C,D) MR effect size for HMGCR and PCSK9 on nonmelanoma skin cancer (ieu-b-4959). (E,F) MR effect size for HMGCR and PCSK9 on psoriasis (ebi-a-GCST90018907). The black dots and lines represent the total estimate and 95% CI of MR analysis by MREgger regression and inverse-variance weighted (IVW) methods. The significance of red lines are MR results of MR-Egger and inverse-variance weighted (IVW) methods. [file Image_1.JPEG]

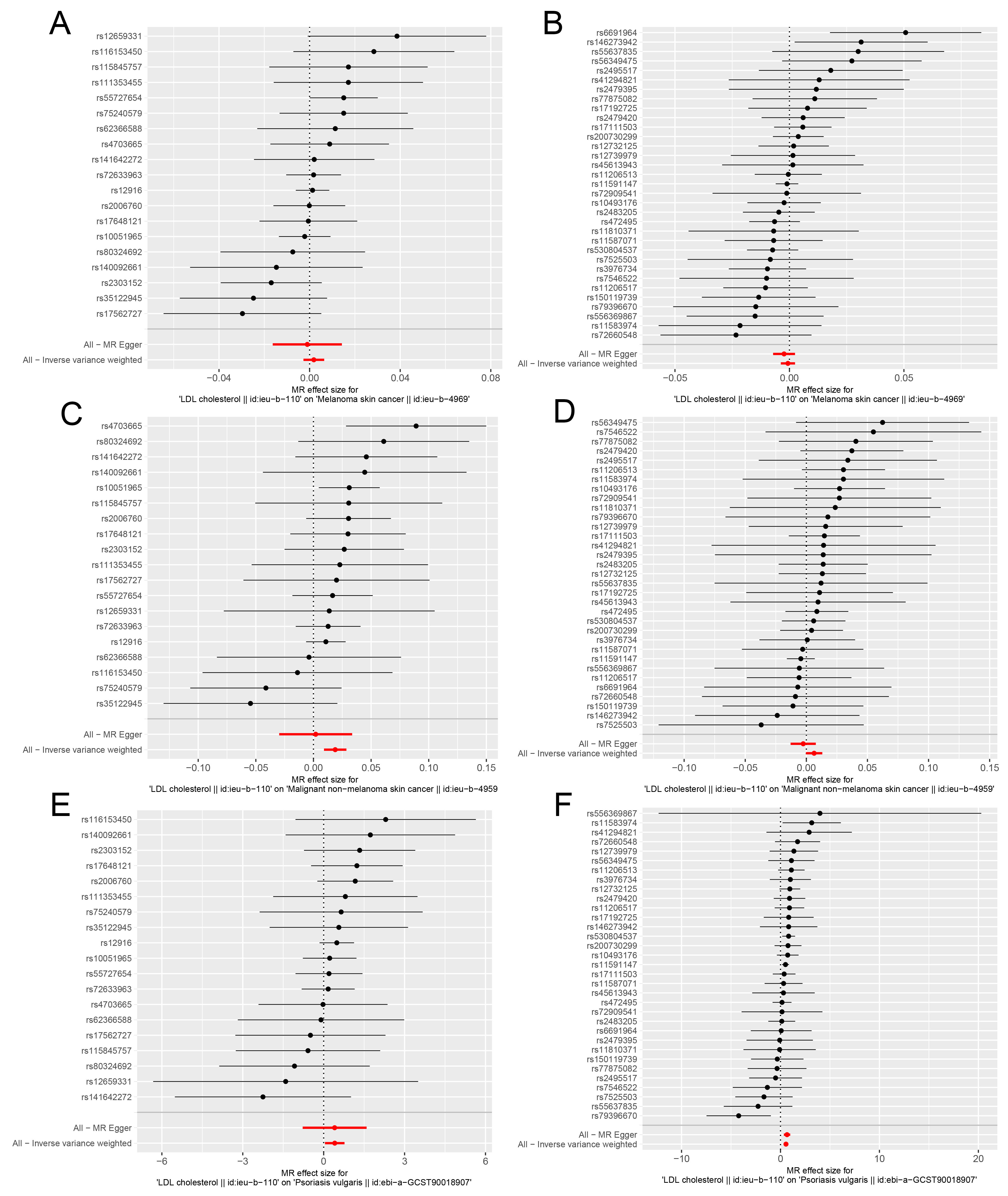

Supplement: SUPPLEMENTARY FIGURE 2 — Validation of the results from the two-sample MR analysis using inverse variance weighted (IVW) method in the ieu-b-110 dataset. (A,B) MR effect size for HMGCR and PCSK9 on melanoma skin cancer (ieu-b-4969). (C,D) MR effect size for HMGCR and PCSK9 on nonmelanoma skin cancer (ieu-b-4959). (E,F) MR effect size for HMGCR and PCSK9 on psoriasis (ebi-a-GCST90018907). The black dots and lines represent the total estimate and 95% CI of MR analysis by MR-Egger regression and IVW methods. The significance of red lines are MR results of MR-Egger and inverse-variance weighted (IVW) methods. [file Image_2.JPEG]
