# Supplementary material for: Causal effects of lipid-lowering drugs on skin diseases: a two-sample Mendelian randomization study
Source: Front Med (Lausanne). 2024 Sep 25;11:1396036. doi: 10.3389/fmed.2024.1396036 (PMC11461303; doi:10.3389/fmed.2024.1396036)
Supplement: Supplementary file 3 [file Table_1.DOCX]

**Table S1** Selection of target genes of lipid-lowering drugs

| **Drugs** | **drugIDs** | **Target Genes** |
| --- | --- | --- |
| Atorvastatin | DB01076 | HMGCR, DPP4, AHR, HDAC2, NR1I3 |
| Alirocumab | DB09302 | PCSK9 |
| Fluvastatin | DB01095 | HMGCR, HDAC2 |
| Lovastatin | DB00227 | HMGCR, ITGAL, HDAC2 |
| Pravastatin sodium | DB00175 | HMGCR, HDAC2 |
| Simvastatin | DB00641 | HMGCR, ITGAL, HDAC2 |
| Rosuvastatin | DB01098 | HMGCR, ITGAL |
