# Supplementary material for: Causal effects of lipid-lowering drugs on skin diseases: a two-sample Mendelian randomization study
Source: Front Med (Lausanne). 2024 Sep 25;11:1396036. doi: 10.3389/fmed.2024.1396036 (PMC11461303; doi:10.3389/fmed.2024.1396036)
Supplement: Supplementary file 4 [file Table_2.DOCX]

**Supplementary Table 2** 7 SNPs in HMGCR in the ieu-b-5089 dataset for melanoma skin cancer

| SNP | Organism | Position | effect_allele.exposure | other_allele.exposure | effect_allele.outcome | other_allele.outcome | beta.exposure | beta.outcome | pval.exposure | pval.outcome |
| --- | --- | --- | --- | --- | --- | --- | --- | --- | --- | --- |
| rs10079346 | Homo sapiens | chr5:75240830 (GRCh38.p14) | A | G | A | G | 0.0392565 | -0.000137518 | 1.90E-34 | 0.56 |
| rs17648121 | Homo sapiens | chr5:75354281 (GRCh38.p14) | T | C | T | C | 0.059432 | -3.40E-05 | 1.00E-10 | 0.96 |
| rs2006760 | Homo sapiens | chr5:75266204 (GRCh38.p14) | G | C | G | C | 0.0318844 | -4.37E-06 | 1.20E-16 | 0.99 |
| rs2303152 | Homo sapiens | chr5:75345882 (GRCh38.p14) | A | G | A | G | 0.0311566 | -0.000564817 | 7.40E-10 | 0.14 |
| rs4704213 | Homo sapiens | chr5:75387075 (GRCh38.p14) | A | G | A | G | 0.05243 | 0.000111706 | 1.50E-28 | 0.75 |
| rs55727654 | Homo sapiens | chr5:75356039 (GRCh38.p14) | A | G | A | G | 0.0373204 | 0.000635354 | 2.30E-17 | 0.049 |
| rs6453131 | Homo sapiens | chr5:75348881 (GRCh38.p14) | G | T | G | T | 0.0565272 | 0.000150402 | 2.30E-69 | 0.53 |
