# Supplementary material for: Causal effects of lipid-lowering drugs on skin diseases: a two-sample Mendelian randomization study
Source: Front Med (Lausanne). 2024 Sep 25;11:1396036. doi: 10.3389/fmed.2024.1396036 (PMC11461303; doi:10.3389/fmed.2024.1396036)
Supplement: Supplementary file 6 [file Table_4.DOCX]

**Supplementary Table 4** 7 SNPs in HMGCR in the ieu-b-5089 dataset for non-melanoma skin cancer

| SNP | Organism | Position | effect_allele.exposure | other_allele.exposure | effect_allele.outcome | other_allele.outcome | beta.exposure | beta.outcome | pval.exposure | pval.outcome |
| --- | --- | --- | --- | --- | --- | --- | --- | --- | --- | --- |
| rs10079346 | Homo sapiens | chr5:75240830 (GRCh38.p14) | A | G | A | G | 0.0392565 | 0.00140491 | 1.90E-34 | 0.01 |
| rs17648121 | Homo sapiens | chr5:75354281 (GRCh38.p14) | T | C | T | C | 0.059432 | 0.00185813 | 1.00E-10 | 0.24 |
| rs2006760 | Homo sapiens | chr5:75266204 (GRCh38.p14) | G | C | G | C | 0.0318844 | 0.00108287 | 1.20E-16 | 0.10 |
| rs2303152 | Homo sapiens | chr5:75345882 (GRCh38.p14) | A | G | A | G | 0.0311566 | 0.000891299 | 7.40E-10 | 0.31 |
| rs4704213 | Homo sapiens | chr5:75387075 (GRCh38.p14) | A | G | A | G | 0.05243 | 0.000678599 | 1.50E-28 | 0.40 |
| rs55727654 | Homo sapiens | chr5:75356039 (GRCh38.p14) | A | G | A | G | 0.0373204 | 0.000698691 | 2.30E-17 | 0.35 |
| rs6453131 | Homo sapiens | chr5:75348881 (GRCh38.p14) | G | T | G | T | 0.0565272 | 0.00102307 | 2.30E-69 | 0.06 |
