# Supplementary material for: Causal effects of lipid-lowering drugs on skin diseases: a two-sample Mendelian randomization study
Source: Front Med (Lausanne). 2024 Sep 25;11:1396036. doi: 10.3389/fmed.2024.1396036 (PMC11461303; doi:10.3389/fmed.2024.1396036)
Supplement: Supplementary file 9 [file Table_7.DOCX]

**Supplementary Table** **7** 16 SNPs in PCSK9 in the ieu-b-5089 dataset for psoriasis

| SNP | Organism | Position | effect_allele.exposure | other_allele.exposure | effect_allele.outcome | other_allele.outcome | beta.exposure | beta.outcome | pval.exposure | pval.outcome |
| --- | --- | --- | --- | --- | --- | --- | --- | --- | --- | --- |
| rs10493176 | Homo sapiens | chr1:55072879 (GRCh38.p14) | G | T | G | T | -0.0577103 | -0.0388 | 7.30E-23 | 0.19 |
| rs11587071 | Homo sapiens | chr1:55057001 (GRCh38.p14) | T | C | T | C | -0.0278058 | -0.0088 | 9.70E-12 | 0.75 |
| rs11591147 | Homo sapiens | chr1:55039974 (GRCh38.p14) | T | G | T | G | -0.343211 | -0.1765 | 1.00E-187 | 0.01 |
| rs12732125 | Homo sapiens | chr1:55004480 (GRCh38.p14) | T | C | T | C | -0.11109 | -0.0977 | 2.70E-24 | 0.08 |
| rs17111503 | Homo sapiens | chr1:55037775 (GRCh38.p14) | G | A | G | A | 0.0421054 | 0.015 | 2.00E-33 | 0.53 |
| rs2483205 | Homo sapiens | chr1:55052643 (GRCh38.p14) | T | C | T | C | -0.0334091 | -0.0038 | 8.00E-26 | 0.85 |
| rs2495500 | Homo sapiens | chr1:55021995 (GRCh38.p14) | T | A | T | A | -0.023115 | -0.0263 | 5.20E-11 | 0.24 |
| rs3976734 | Homo sapiens | chr1:55024287 (GRCh38.p14) | G | A | G | A | -0.02817 | -0.0297 | 3.50E-16 | 0.35 |
| rs472495 | Homo sapiens | chr1:55055640 (GRCh38.p14) | T | G | T | G | 0.0447232 | 0.0066 | 1.20E-43 | 0.75 |
| rs4927191 | Homo sapiens | chr1:55026029 (GRCh38.p14) | C | T | C | T | -0.0429521 | -0.0368 | 1.60E-34 | 0.10 |
| rs505151 | Homo sapiens | chr1:55063514 (GRCh38.p14) | A | G | A | G | -0.073956 | -0.0614 | 5.30E-17 | 0.22 |
| rs530804537 | Homo sapiens | chr1:55117537 (GRCh38.p14) | A | G | A | G | -0.201771 | -0.1583 | 3.10E-43 | 0.01 |
| rs6691964 | Homo sapiens | chr1:54968305 (GRCh38.p14) | A | G | A | G | -0.031203 | -0.0017 | 7.00E-09 | 0.96 |
| rs72660539 | Homo sapiens | chr1:55029233 (GRCh38.p14) | A | G | A | G | 0.0366186 | 0.0014 | 2.80E-15 | 0.97 |
| rs7543163 | Homo sapiens | chr1:55049808 (GRCh38.p14) | T | C | T | C | 0.034815 | 0.0337 | 8.10E-28 | 0.12 |
| rs77875082 | Homo sapiens | chr1:55019369 (GRCh38.p14) | A | G | A | G | 0.053705 | -0.0167 | 2.70E-09 | 0.82 |
