# Supplementary material for: Causal effects of lipid-lowering drugs on skin diseases: a two-sample Mendelian randomization study
Source: Front Med (Lausanne). 2024 Sep 25;11:1396036. doi: 10.3389/fmed.2024.1396036 (PMC11461303; doi:10.3389/fmed.2024.1396036)
Supplement: Supplementary file 10 [file Table_8.DOCX]

**Supplementary Table 8** 19 SNPs in HMGCR in the ieu-b-110 dataset for melanoma skin cancer

| SNP | Organism | Position | effect_allele.exposure | other_allele.exposure | effect_allele.outcome | other_allele.outcome | beta.exposure | beta.outcome | pval.exposure | pval.outcome |
| --- | --- | --- | --- | --- | --- | --- | --- | --- | --- | --- |
| rs10051965 | Homo sapiens | chr5:75264662 (GRCh38.p14) | T | C | T | C | 0.0410063 | -8.88E-05 | 5.40E-80 | 0.71 |
| rs111353455 | Homo sapiens | chr5:75328124 (GRCh38.p14) | A | G | A | G | 0.0243909 | 0.000417606 | 6.00E-11 | 0.31 |
| rs115845757 | Homo sapiens | chr5:75267875 (GRCh38.p14) | A | G | A | G | 0.048608 | 0.000833775 | 6.10E-10 | 0.34 |
| rs116153450 | Homo sapiens | chr5:75433608 (GRCh38.p14) | A | C | A | C | -0.0303618 | -0.000861529 | 1.20E-09 | 0.12 |
| rs12659331 | Homo sapiens | chr5:75461832 (GRCh38.p14) | C | A | C | A | 0.0251785 | 0.000971473 | 4.20E-08 | 0.05 |
| rs12916 | Homo sapiens | chr5:75360714 (GRCh38.p14) | C | T | C | T | 0.0621175 | 8.11E-05 | 1.70E-187 | 0.73 |
| rs140092661 | Homo sapiens | chr5:75386775 (GRCh38.p14) | T | A | T | A | 0.0329927 | -0.000483791 | 1.50E-08 | 0.45 |
| rs141642272 | Homo sapiens | chr5:75319384 (GRCh38.p14) | C | G | C | G | 0.0532822 | 0.000108955 | 3.70E-16 | 0.88 |
| rs17562727 | Homo sapiens | chr5:75386649 (GRCh38.p14) | C | T | C | T | 0.0394972 | -0.00117098 | 5.30E-10 | 0.09 |
| rs17648121 | Homo sapiens | chr5:75354281 (GRCh38.p14) | T | C | T | C | 0.0619849 | -3.40E-05 | 1.40E-23 | 0.96 |
| rs2006760 | Homo sapiens | chr5:75266204 (GRCh38.p14) | G | C | G | C | 0.03556 | -4.37E-06 | 3.00E-42 | 0.99 |
| rs2303152 | Homo sapiens | chr5:75345882 (GRCh38.p14) | A | G | A | G | 0.0333589 | -0.000564817 | 4.40E-22 | 0.14 |
| rs35122945 | Homo sapiens | chr5:75314468 (GRCh38.p14) | C | A | C | A | -0.0281057 | 0.000696519 | 3.30E-11 | 0.14 |
| rs4703665 | Homo sapiens | chr5:75307073 (GRCh38.p14) | C | T | C | T | 0.0244938 | 0.00021723 | 1.90E-16 | 0.51 |
| rs55727654 | Homo sapiens | chr5:75356039 (GRCh38.p14) | A | G | A | G | 0.042154 | 0.000635354 | 6.90E-47 | 0.05 |
| rs62366588 | Homo sapiens | chr5:75369162 (GRCh38.p14) | A | C | A | C | -0.0271295 | -0.000307675 | 3.70E-10 | 0.52 |
| rs72633963 | Homo sapiens | chr5:75335004 (GRCh38.p14) | A | G | A | G | 0.0564278 | 0.000100075 | 4.90E-71 | 0.77 |
| rs75240579 | Homo sapiens | chr5:75328659 (GRCh38.p14) | T | C | T | C | -0.0372115 | -0.00056018 | 2.20E-14 | 0.30 |
| rs80324692 | Homo sapiens | chr5:75421936 (GRCh38.p14) | T | C | T | C | -0.0260509 | 0.000193545 | 1.40E-11 | 0.65 |
