# Supplementary material for: Causal effects of lipid-lowering drugs on skin diseases: a two-sample Mendelian randomization study
Source: Front Med (Lausanne). 2024 Sep 25;11:1396036. doi: 10.3389/fmed.2024.1396036 (PMC11461303; doi:10.3389/fmed.2024.1396036)
Supplement: Supplementary file 11 [file Table_9.DOCX]

**Supplementary Table 9** 33 SNPs in PCSK9 in the ieu-b-110 dataset for melanoma skin cancer

| SNP | Organism | Position | effect_allele.exposure | other_allele.exposure | effect_allele.outcome | other_allele.outcome | beta.exposure | beta.outcome | pval.exposure | pval.outcome |
| --- | --- | --- | --- | --- | --- | --- | --- | --- | --- | --- |
| rs10493176 | Homo sapiens | chr1:55072879 (GRCh38.p14) | G | T | G | T | -0.0531381 | 0.000124006 | 2.60E-41 | 0.78 |
| rs11206513 | Homo sapiens | chr1:55041976 (GRCh38.p14) | T | C | T | C | 0.0316517 | -1.58E-05 | 3.20E-49 | 0.95 |
| rs11206517 | Homo sapiens | chr1:55060755 (GRCh38.p14) | G | T | G | T | 0.0680285 | -0.000710105 | 1.00E-31 | 0.27 |
| rs11583974 | Homo sapiens | chr1:55086045 (GRCh38.p14) | A | G | A | G | 0.0314531 | -0.000678345 | 1.20E-09 | 0.23 |
| rs11587071 | Homo sapiens | chr1:55057001 (GRCh38.p14) | T | C | T | C | -0.0282322 | 0.000194661 | 5.30E-24 | 0.53 |
| rs11591147 | Homo sapiens | chr1:55039974 (GRCh38.p14) | T | G | T | G | -0.348456 | 0.000372303 | 1.00E-200 | 0.67 |
| rs11810371 | Homo sapiens | chr1:55031188 (GRCh38.p14) | A | G | A | G | -0.0294547 | 0.000202859 | 6.40E-09 | 0.72 |
| rs12732125 | Homo sapiens | chr1:55004480 (GRCh38.p14) | T | C | T | C | -0.10344 | -0.000190742 | 1.00E-44 | 0.81 |
| rs12739979 | Homo sapiens | chr1:55030975 (GRCh38.p14) | T | C | T | C | -0.0202563 | -3.10E-05 | 1.50E-15 | 0.91 |
| rs146273942 | Homo sapiens | chr1:54988168 (GRCh38.p14) | A | G | A | G | -0.0538858 | -0.00168765 | 8.70E-14 | 0.04 |
| rs150119739 | Homo sapiens | chr1:55055265 (GRCh38.p14) | A | G | A | G | 0.0452728 | -0.000607429 | 3.20E-18 | 0.29 |
| rs17111503 | Homo sapiens | chr1:55037775 (GRCh38.p14) | G | A | G | A | 0.0406795 | 0.000237931 | 1.00E-66 | 0.36 |
| rs17192725 | Homo sapiens | chr1:55030458 (GRCh38.p14) | A | G | A | G | 0.0305717 | 0.000241551 | 6.40E-17 | 0.55 |
| rs200730299 | Homo sapiens | chr1:55026180 (GRCh38.p14) | C | A | C | A | -0.0543492 | -0.000209986 | 5.10E-85 | 0.49 |
| rs2479395 | Homo sapiens | chr1:55018909 (GRCh38.p14) | C | T | C | T | 0.0125674 | 0.000147607 | 1.50E-08 | 0.55 |
| rs2479420 | Homo sapiens | chr1:55026517 (GRCh38.p14) | T | C | T | C | -0.0283879 | -0.00017007 | 9.90E-33 | 0.52 |
| rs2483205 | Homo sapiens | chr1:55052643 (GRCh38.p14) | T | C | T | C | -0.0295845 | 0.000137232 | 2.90E-43 | 0.56 |
| rs2495517 | Homo sapiens | chr1:54983169 (GRCh38.p14) | G | A | G | A | 0.0177548 | 0.000320522 | 5.80E-12 | 0.26 |
| rs3976734 | Homo sapiens | chr1:55024287 (GRCh38.p14) | G | A | G | A | -0.0297494 | 0.000285806 | 1.10E-37 | 0.26 |
| rs41294821 | Homo sapiens | chr1:55047510 (GRCh38.p14) | T | C | T | C | -0.0386615 | -0.000502416 | 4.20E-08 | 0.52 |
| rs45613943 | Homo sapiens | chr1:55052949 (GRCh38.p14) | C | T | C | T | -0.0340702 | -5.05E-05 | 2.60E-12 | 0.93 |
| rs472495 | Homo sapiens | chr1:55055640 (GRCh38.p14) | T | G | T | G | 0.0425743 | -0.000276859 | 7.30E-85 | 0.25 |
| rs530804537 | Homo sapiens | chr1:55117537 (GRCh38.p14) | A | G | A | G | -0.192336 | 0.00141228 | 7.80E-83 | 0.20 |
| rs556369867 | Homo sapiens | chr1:55025462 (GRCh38.p14) | T | C | T | C | 0.0175746 | -0.00026348 | 4.80E-13 | 0.33 |
| rs55637835 | Homo sapiens | chr1:55000630 (GRCh38.p14) | T | C | T | C | -0.0187129 | -0.000561915 | 8.40E-09 | 0.12 |
| rs56349475 | Homo sapiens | chr1:55110429 (GRCh38.p14) | C | T | C | T | -0.0475957 | -0.0012982 | 1.40E-12 | 0.08 |
| rs6691964 | Homo sapiens | chr1:54968305 (GRCh38.p14) | A | G | A | G | -0.0234719 | -0.00119124 | 5.80E-11 | 0.003 |
| rs72660548 | Homo sapiens | chr1:55035305 (GRCh38.p14) | G | C | G | C | 0.0509816 | -0.00119008 | 5.50E-11 | 0.17 |
| rs72909541 | Homo sapiens | chr1:55028628 (GRCh38.p14) | T | C | T | C | -0.0334061 | 3.89E-05 | 2.70E-11 | 0.94 |
| rs7525503 | Homo sapiens | chr1:55056885 (GRCh38.p14) | T | G | T | G | 0.0454642 | -0.000379683 | 2.00E-09 | 0.65 |
| rs7546522 | Homo sapiens | chr1:55051040 (GRCh38.p14) | T | C | T | C | -0.0168117 | 0.000167784 | 1.20E-08 | 0.61 |
| rs77875082 | Homo sapiens | chr1:55019369 (GRCh38.p14) | A | G | A | G | 0.0481535 | 0.000533112 | 1.80E-15 | 0.42 |
| rs79396670 | Homo sapiens | chr1:55122469 (GRCh38.p14) | A | G | A | G | -0.0336489 | 0.000494593 | 2.10E-09 | 0.42 |
