# Supplementary material for: Causal effects of lipid-lowering drugs on skin diseases: a two-sample Mendelian randomization study
Source: Front Med (Lausanne). 2024 Sep 25;11:1396036. doi: 10.3389/fmed.2024.1396036 (PMC11461303; doi:10.3389/fmed.2024.1396036)
Supplement: Supplementary file 12 [file Table_10.DOCX]

**Supplementary Table 10** 19 SNPs in HMGCR in the ieu-b-110 dataset for non-melanoma skin cancer

| SNP | Organism | Position | effect_allele.exposure | other_allele.exposure | effect_allele.outcome | other_allele.outcome | beta.exposure | beta.outcome | pval.exposure | pval.outcome |
| --- | --- | --- | --- | --- | --- | --- | --- | --- | --- | --- |
| rs10051965 | Homo sapiens | chr5:75264662 (GRCh38.p14) | T | C | T | C | 0.0410063 | 0.0012734 | 5.40E-80 | 0.02 |
| rs111353455 | Homo sapiens | chr5:75328124 (GRCh38.p14) | A | G | A | G | 0.0243909 | 0.000557785 | 6.00E-11 | 0.56 |
| rs115845757 | Homo sapiens | chr5:75267875 (GRCh38.p14) | A | G | A | G | 0.048608 | 0.00148814 | 6.10E-10 | 0.46 |
| rs116153450 | Homo sapiens | chr5:75433608 (GRCh38.p14) | A | C | A | C | -0.0303618 | 0.000420327 | 1.20E-09 | 0.74 |
| rs12659331 | Homo sapiens | chr5:75461832 (GRCh38.p14) | C | A | C | A | 0.0251785 | 0.000344829 | 4.20E-08 | 0.77 |
| rs12916 | Homo sapiens | chr5:75360714 (GRCh38.p14) | C | T | C | T | 0.0621175 | 0.000668888 | 1.70E-187 | 0.22 |
| rs140092661 | Homo sapiens | chr5:75386775 (GRCh38.p14) | T | A | T | A | 0.0329927 | 0.00146749 | 1.50E-08 | 0.32 |
| rs141642272 | Homo sapiens | chr5:75319384 (GRCh38.p14) | C | G | C | G | 0.0532822 | 0.0024437 | 3.70E-16 | 0.14 |
| rs17562727 | Homo sapiens | chr5:75386649 (GRCh38.p14) | C | T | C | T | 0.0394972 | 0.000789612 | 5.30E-10 | 0.63 |
| rs17648121 | Homo sapiens | chr5:75354281 (GRCh38.p14) | T | C | T | C | 0.0619849 | 0.00185813 | 1.40E-23 | 0.24 |
| rs2006760 | Homo sapiens | chr5:75266204 (GRCh38.p14) | G | C | G | C | 0.03556 | 0.00108287 | 3.00E-42 | 0.10 |
| rs2303152 | Homo sapiens | chr5:75345882 (GRCh38.p14) | A | G | A | G | 0.0333589 | 0.000891299 | 4.40E-22 | 0.31 |
| rs35122945 | Homo sapiens | chr5:75314468 (GRCh38.p14) | C | A | C | A | -0.0281057 | 0.00153586 | 3.30E-11 | 0.16 |
| rs4703665 | Homo sapiens | chr5:75307073 (GRCh38.p14) | C | T | C | T | 0.0244938 | 0.00218146 | 1.90E-16 | 0.004 |
| rs55727654 | Homo sapiens | chr5:75356039 (GRCh38.p14) | A | G | A | G | 0.042154 | 0.000698691 | 6.90E-47 | 0.35 |
| rs62366588 | Homo sapiens | chr5:75369162 (GRCh38.p14) | A | C | A | C | -0.0271295 | 0.000105236 | 3.70E-10 | 0.92 |
| rs72633963 | Homo sapiens | chr5:75335004 (GRCh38.p14) | A | G | A | G | 0.0564278 | 0.000718774 | 4.90E-71 | 0.37 |
| rs75240579 | Homo sapiens | chr5:75328659 (GRCh38.p14) | T | C | T | C | -0.0372115 | 0.00153524 | 2.20E-14 | 0.22 |
| rs80324692 | Homo sapiens | chr5:75421936 (GRCh38.p14) | T | C | T | C | -0.0260509 | -0.00158809 | 1.40E-11 | 0.11 |
