# Supplementary material for: Causal effects of lipid-lowering drugs on skin diseases: a two-sample Mendelian randomization study
Source: Front Med (Lausanne). 2024 Sep 25;11:1396036. doi: 10.3389/fmed.2024.1396036 (PMC11461303; doi:10.3389/fmed.2024.1396036)
Supplement: Supplementary file 14 [file Table_12.DOCX]

**Supplementary Table 12** 19 SNPs in HMGCR in the ieu-b-110 dataset for psoriasis

| SNP | Organism | Position | effect_allele.exposure | other_allele.exposure | effect_allele.outcome | other_allele.outcome | beta.exposure | beta.outcome | pval.exposure | pval.outcome |
| --- | --- | --- | --- | --- | --- | --- | --- | --- | --- | --- |
| rs10051965 | Homo sapiens | chr5:75264662 (GRCh38.p14) | T | C | T | C | 0.0410063 | 0.0091 | 5.40E-80 | 0.66 |
| rs111353455 | Homo sapiens | chr5:75328124 (GRCh38.p14) | A | G | A | G | 0.0243909 | 0.0196 | 6.00E-11 | 0.56 |
| rs115845757 | Homo sapiens | chr5:75267875 (GRCh38.p14) | A | G | A | G | 0.048608 | -0.0283 | 6.10E-10 | 0.67 |
| rs116153450 | Homo sapiens | chr5:75433608 (GRCh38.p14) | A | C | A | C | -0.0303618 | -0.0698 | 1.20E-09 | 0.18 |
| rs12659331 | Homo sapiens | chr5:75461832 (GRCh38.p14) | C | A | C | A | 0.0251785 | -0.0355 | 4.20E-08 | 0.57 |
| rs12916 | Homo sapiens | chr5:75360714 (GRCh38.p14) | C | T | C | T | 0.0621175 | 0.0302 | 1.70E-187 | 0.14 |
| rs140092661 | Homo sapiens | chr5:75386775 (GRCh38.p14) | T | A | T | A | 0.0329927 | 0.0571 | 1.50E-08 | 0.28 |
| rs141642272 | Homo sapiens | chr5:75319384 (GRCh38.p14) | C | G | C | G | 0.0532822 | -0.12 | 3.70E-16 | 0.18 |
| rs17562727 | Homo sapiens | chr5:75386649 (GRCh38.p14) | C | T | C | T | 0.0394972 | -0.0194 | 5.30E-10 | 0.73 |
| rs17648121 | Homo sapiens | chr5:75354281 (GRCh38.p14) | T | C | T | C | 0.0619849 | 0.0763 | 1.40E-23 | 0.16 |
| rs2006760 | Homo sapiens | chr5:75266204 (GRCh38.p14) | G | C | G | C | 0.03556 | 0.0416 | 3.00E-42 | 0.10 |
| rs2303152 | Homo sapiens | chr5:75345882 (GRCh38.p14) | A | G | A | G | 0.0333589 | 0.0445 | 4.40E-22 | 0.20 |
| rs35122945 | Homo sapiens | chr5:75314468 (GRCh38.p14) | C | A | C | A | -0.0281057 | -0.0158 | 3.30E-11 | 0.67 |
| rs4703665 | Homo sapiens | chr5:75307073 (GRCh38.p14) | C | T | C | T | 0.0244938 | -6.00E-04 | 1.90E-16 | 0.98 |
| rs55727654 | Homo sapiens | chr5:75356039 (GRCh38.p14) | A | G | A | G | 0.042154 | 0.0083 | 6.90E-47 | 0.76 |
| rs62366588 | Homo sapiens | chr5:75369162 (GRCh38.p14) | A | C | A | C | -0.0271295 | 0.0027 | 3.70E-10 | 0.95 |
| rs72633963 | Homo sapiens | chr5:75335004 (GRCh38.p14) | A | G | A | G | 0.0564278 | 0.0094 | 4.90E-71 | 0.74 |
| rs75240579 | Homo sapiens | chr5:75328659 (GRCh38.p14) | T | C | T | C | -0.0372115 | -0.0241 | 2.20E-14 | 0.67 |
| rs80324692 | Homo sapiens | chr5:75421936 (GRCh38.p14) | T | C | T | C | -0.0260509 | 0.0282 | 1.40E-11 | 0.45 |
